# Supplementary material for: The transcription factor hepatocyte nuclear factor 4A acts in the intestine to promote white adipose tissue energy storage
Source: Nat Commun. 2022 Jan 11;13:224. doi: 10.1038/s41467-021-27934-w (PMC8752770; doi:10.1038/s41467-021-27934-w)
Supplement: Supplementary file 1 — Supplementary Information [file 41467_2021_27934_MOESM1_ESM.pdf]

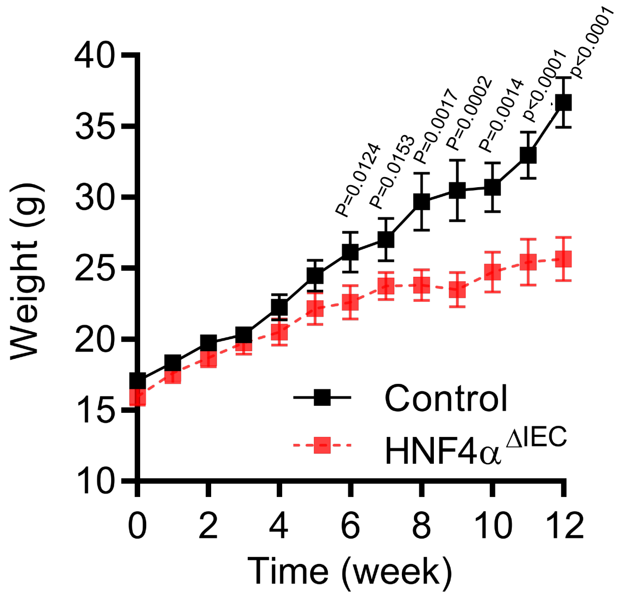

Supplemental Fig. S1: **Female mice lacking intestinal HNF4A were resistant to HFD-induced obesity.** Weight curve of weaned mice fed an HFD for 12 weeks for control (black squares, n = 7) and HNF4A$^{\Delta IEC}$ mutant (red squares, n = 8) mice. Statistical comparisons were performed using two-way ANOVA followed by uncorrected Fisher's LSD test. Data are presented as mean values +/- SEM. Source data are provided as a Source Data file.

a

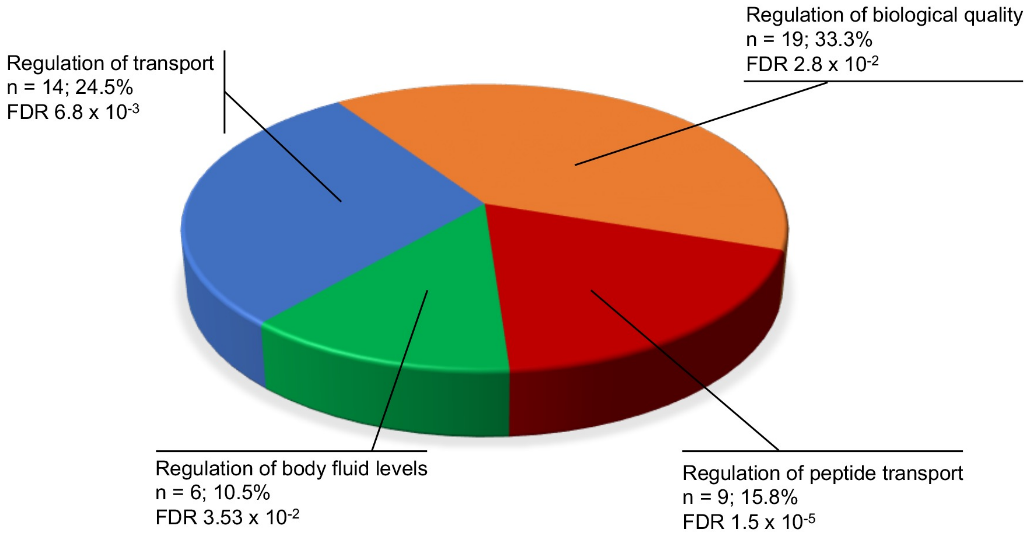

b

Gene name	Gene symbol	Protein Class
Fibrinogen alpha chain	Fga	
Carbonic anhydrase 2	Car2	
C-C motif chemokine 5	Cd5	cytokine
Gastric inhibitory polypeptide	GIP	hormone
Hepatocyte nuclear factor 4-a	Hnf4a	Zinc finger nuclear receptor
Protein FAM3D	Famd3	Antimicrobial response protein
Immunoglobulin-like domain containing receptor 1	Ildr1	Immunoglobulin receptor superfamily
Fibrinogen beta chain	Fgb	Intercellular signal molecule
Fibrinogen gamma chain	Fgg	Intercellular signal molecule

Supplemental Fig. S2: **Transcriptomic analysis of the jejunum of HNF4A$^{\Delta IEC}$ mutants under HFD. a,** Biological functions associated with transcriptomic changes in the jejunum of HNF4A$^{\Delta IEC}$ mutants. **b**, List of genes significantly modulated in the jejunum of HNF4A$^{\Delta IEC}$ mutants and associated with regulation of peptide transport.

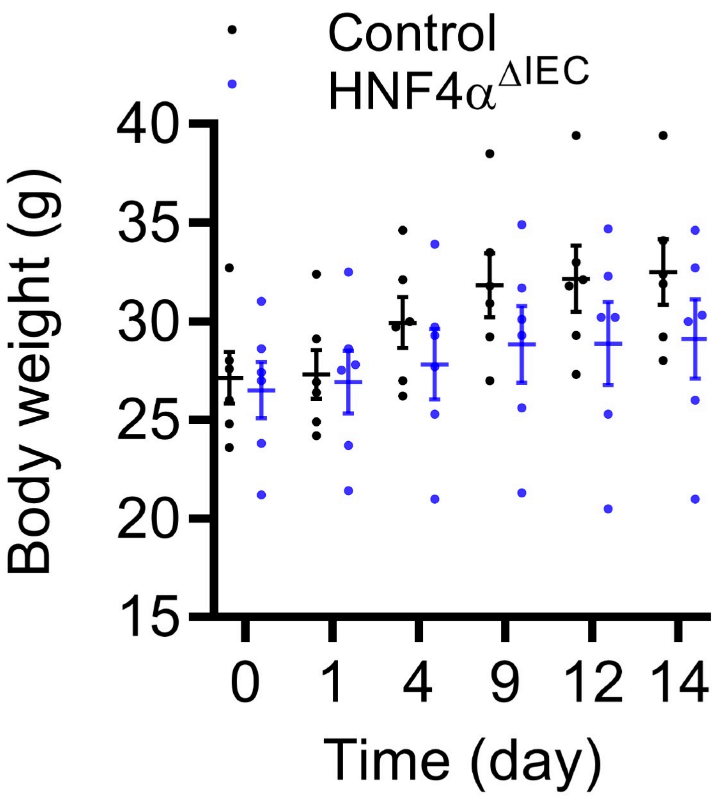

Supplemental Fig. S3: **Individual weight values of control (black points) and HNF4A$^{\Delta IEC}$ mutant (blue points) mice fed an HFD as monitored for 2 weeks (n = 6).** Data are presented as mean values +/- SEM.
