# Supplementary material for: The transcription factor hepatocyte nuclear factor 4A acts in the intestine to promote white adipose tissue energy storage
Source: Nat Commun. 2022 Jan 11;13:224. doi: 10.1038/s41467-021-27934-w (PMC8752770; doi:10.1038/s41467-021-27934-w)
Supplement: Supplementary file 3 — Reporting Summary [file 41467_2021_27934_MOESM3_ESM.pdf]

nature portfolio

Corresponding author(s): *Double-blind peer review submissions: write DBPR and your manuscript number here instead of author names.*

Last updated by author(s): *YYYY-MM-DD*

Reporting Summary

Nature Portfolio wishes to improve the reproducibility of the work that we publish. This form provides structure for consistency and transparency in reporting. For further information on Nature Portfolio policies, see our Editorial Policies and the Editorial Policy Checklist.

Statistics

For all statistical analyses, confirm that the following items are present in the figure legend, table legend, main text, or Methods section.

n/a	Confirmed	
☐	☒	The exact sample size (*n*) for each experimental group/condition, given as a discrete number and unit of measurement
☐	☒	A statement on whether measurements were taken from distinct samples or whether the same sample was measured repeatedly
☐	☒	The statistical test(s) used AND whether they are one- or two-sided<br>*Only common tests should be described solely by name; describe more complex techniques in the Methods section.*
☒	☐	A description of all covariates tested
☐	☒	A description of any assumptions or corrections, such as tests of normality and adjustment for multiple comparisons
☐	☒	A full description of the statistical parameters including central tendency (e.g. means) or other basic estimates (e.g. regression coefficient) AND variation (e.g. standard deviation) or associated estimates of uncertainty (e.g. confidence intervals)
☐	☒	For null hypothesis testing, the test statistic (e.g. *F*, *t*, *r*) with confidence intervals, effect sizes, degrees of freedom and *P* value noted<br>*Give P values as exact values whenever suitable.*
☒	☐	For Bayesian analysis, information on the choice of priors and Markov chain Monte Carlo settings
☒	☐	For hierarchical and complex designs, identification of the appropriate level for tests and full reporting of outcomes
☒	☐	Estimates of effect sizes (e.g. Cohen's *d*, Pearson's *r*), indicating how they were calculated

Our web collection on statistics for biologists contains articles on many of the points above.

Software and code

Policy information about availability of computer code

Data collection	Quality control and adapter trimming of RNA-seq libraries were performed using Trim Galore version 0.6.4_dev (https://www.bioinformatics.babraham.ac.uk/projects/trim_galore/). Reads were aligned with Rsubread version 2.6.2 54 against mouse genome version GRCm38 in pair-end mode. Low-count genes were filtered with DEseq2 version 1.32.0 and differential expression analysis was performed using edgeR version 3.34.0. Amide software v.1.0.5 was used for PET/CT acquisition. Indirect calorimetry raw data were processed using ExpeData v. 1.8.4 (Sable Systems, Las Vegas, NV).
Data analysis	Adipocyte histomorphology was analyzed using CellProfiler (https://cellprofiler.org). Raw cel files from affymetrix microarrays were analyzed using Transcriptome Analysis Console (TAC) software (ThermoFisher Scientific). GSEA was used for transcriptome analyses (http://www.gseamsigdb.org/gsea/index.jsp). GO transcriptomic analyses were also analysed with DAVID version 6.8 (https://david.ncifcrf.gov). Statistical analyses were performed and graphics were produced using GraphPad Prism 8 software.

For manuscripts utilizing custom algorithms or software that are central to the research but not yet described in published literature, software must be made available to editors and reviewers. We strongly encourage code deposition in a community repository (e.g. GitHub). See the Nature Portfolio guidelines for submitting code & software for further information.

Data

Policy information about availability of data

All manuscripts must include a data availability statement. This statement should provide the following information, where applicable:

- Accession codes, unique identifiers, or web links for publicly available datasets
- A description of any restrictions on data availability
- For clinical datasets or third party data, please ensure that the statement adheres to our policy

Microarray data are accessible through GEO series accession number GSE147105. RNA-seq data are accessible through GEO series accession number (GSE189439). The website for GSEA is available on https://www.gsea-msigdb.org/gsea/index.jsp.

Field-specific reporting

Please select the one below that is the best fit for your research. If you are not sure, read the appropriate sections before making your selection.

☒ Life sciences ☐ Behavioural & social sciences ☐ Ecological, evolutionary & environmental sciences

For a reference copy of the document with all sections, see nature.com/documents/nr-reporting-summary-flat.pdf

Life sciences study design

All studies must disclose on these points even when the disclosure is negative.

Sample size	No sample-size calculation was performed. The sample sizes were chosen accordingly to what is done from comparable studies.
Data exclusions	No data were excluded from this study.
Replication	All attemps at replication were succesfull.
Randomization	Mutant and control mice were randomly distributed into treatment or non-treatment groups.
Blinding	Investigators were blinded to group allocation during data collection.

Reporting for specific materials, systems and methods

We require information from authors about some types of materials, experimental systems and methods used in many studies. Here, indicate whether each material, system or method listed is relevant to your study. If you are not sure if a list item applies to your research, read the appropriate section before selecting a response.

Materials & experimental systems

n/a	Involved in the study	
☐	☒	Antibodies
☐	☒	Eukaryotic cell lines
☒	☐	Palaeontology and archaeology
☐	☒	Animals and other organisms
☒	☐	Human research participants
☒	☐	Clinical data
☒	☐	Dual use research of concern

Methods

n/a	Involved in the study	
☒	☐	ChIP-seq
☒	☐	Flow cytometry
☒	☐	MRI-based neuroimaging

Antibodies

Antibodies used	anti-ADFP (1:500, abcam, Ab52356).
Validation	This antibody reacts with mouse and human epitopes and was validated for IHC by abcam cie (https://www.abcam.com/adfp-antibody-ab52356.html). As stated on their datasheets, every batches are confirmed for specificity through extensive validation.

Eukaryotic cell lines

Policy information about cell lines

Cell line source(s)	ATCC

Authentication	Used within 1 year of acquisition. No authentication was done after purchasing the cell line.
Mycoplasma contamination	The cell line was negative from mycoplasma contamination.
Commonly misidentified lines (See ICLAC register)	Not applicable

Animals and other organisms

Policy information about studies involving animals; ARRIVE guidelines recommended for reporting animal research

Laboratory animals	C57BL/6 female and male mice aged between 3 to 6 months
Wild animals	This study did not involve wild animals
Field-collected samples	This study did not involve samples collected from the field
Ethics oversight	All procedures were approved by the Institutional Animal Research Review Committee of the Université de Sherbrooke (approval 102-18)

Note that full information on the approval of the study protocol must also be provided in the manuscript.
